# Serum Levels of Asymmetric Dimethylarginine and Apelin as Potential Markers of Vascular Endothelial Dysfunction in Early Rheumatoid Arthritis

**DOI:** 10.1155/2012/347268

**Published:** 2012-08-07

**Authors:** Manuela Di Franco, Francesca Romana Spinelli, Alessio Metere, Maria Chiara Gerardi, Virginia Conti, Francesca Boccalini, Cristina Iannuccelli, Francesco Ciciarello, Luciano Agati, Guido Valesini

**Affiliations:** ^1^Department of Internal Medicine and Medical Specialities, Sapienza University of Rome, Viale Del Policlinico 155, 00161 Rome, Italy; ^2^Section of Biomarkers in Degenerative Diseases, Department of Cell Biology and Neurosciences, Istituto Superiore di Sanità, Viale Regina Elena 299, 00161 Rome, Italy; ^3^Department of Cardiovascular and Respiratory Sciences, Sapienza University of Rome, 00161 Rome, Italy

## Abstract

*Objectives*. Impaired endothelial function represents the early stage of atherosclerosis, which is typically associated with systemic inflammatory diseases like rheumatoid arthritis (RA). As modulators of endothelial nitric oxide synthase expression, asymmetric-dimethylarginine (ADMA) and apelin might be measured in the blood of RA patients to detect early atherosclerotic changes. We conducted a prospective, case-control study to investigate serum ADMA and apelin profiles of patients with early-stage RA (ERA) before and after disease-modifying antirheumatic drug (DMARD) therapy. *Methods*. We enrolled 20 consecutively diagnosed, treatment-naïve patients with ERA and 20 matched healthy controls. Serum ADMA and apelin levels and the 28-joint disease activity scores (DAS28) were assessed before and after 12 months of DMARDs treatment. All patients underwent ultrasonographic assessment for intima-media tickness (IMT) evaluation. *Results*. In the ERA group, ADMA serum levels were significantly higher than controls at baseline (*P* = 0.007) and significantly decreased after treatment (*P* = 0.012 versus controls). Baseline serum apelin levels were significantly decreased in this group (*P* = 0.0001 versus controls), but they were not significantly altered by treatment. IMT did not show significant changes. *Conclusions*. ERA is associated with alterations of serum ADMA and apelin levels, which might be used as biomarkers to detect early endothelial dysfunction in these patients.

## 1. Introduction

Rheumatoid arthritis (RA) is a systemic inflammatory disease associated with increased cardiovascular morbidity and mortality [1–5]. The erosive joint damage occurs during the first 2 years of the disease [6, 7], and prompt intervention during this phase may slow the progression to chronic disability [8–10]. Mortality related to cardiovascular disease (CVD) is also increased during the early years of the disease, with a standardized mortality ratio SMR of 1.49 [11]. Therefore, comprehensive care of patients with RA implies prevention and treatment not only of joint damage but also of co-morbidities, particularly cardiovascular disease, which is the cause of 40%–50% of the deaths in this population [4, 5].

Evidence of subclinical CVD has been demonstrated in patients with early RA (ERA) [12]. These patients have also been found to have a higher prevalence of atherosclerotic plaques, increased intima-media thickness (IMT) of the carotid arteries [13, 14], and significantly impaired endothelial function compared with controls [15]. The high frequency in RA patients of risk factors like smoking, dyslipidemia, hypertension, diabetes mellitus, and increased body mass index accounts only partially for their high cardiovascular morbidity and mortality [16, 17]. The accelerated atherosclerosis observed in these patients seems to be due to systemic inflammatory processes [18]. Rheumatoid synovia and atherosclerotic plaques have proinflammatory endothelial phenotypes represented by expression of the same adhesion molecules and cytokines [19].

The availability of a marker of endothelial dysfunction would facilitate the stratification of patients with ERA according to their cardiovascular risk. Increased formation of nitric oxide (NO) has been shown to improve vascular function, attenuate leukocyte adhesion to endothelial cells, inhibit platelet aggregation, and modulate smooth muscle proliferation [20]. In the present study, we examined 2 endogenous regulators of NO production as candidate markers of endothelial function. The first, asymmetric-dimethylarginine (ADMA), is an L-arginine analogue that inhibits endothelial NO synthase (eNOS). Elevated ADMA levels are an independent risk factor for endothelial dysfunction, and they have been associated with hypertension, diabetes, hypercholesterolemia, renal failure, and atherosclerosis in both experimental models and humans [21]. Plasma levels of ADMA are increased in a variety of conditions linked with increased risk of CVD. Higher than normal levels have also been found in patients with established cardiovascular diseases [21] and more recently in RA patients as well [22, 23]. The second potential biomarker we assessed is apelin, a recently described peptide that is known to be produced by several cell types. It causes endothelium-dependent vasorelaxation by triggering the release of NO [24]. This effect is almost completely abolished by the eNOS inhibitor, NG-nitro-L-arginine methyl ester, which indicates that apelin may exert its vasorelaxant effects by activating the eNOS pathway [25]. 

The aim of this case-control study was to evaluate serum levels of these two molecules in ERA patients at the time of diagnosis and to determine whether and how these levels are changed by disease-modifying antirheumatic drug (DMARDs) therapy.

## 2. Patients and Methods

### 2.1. Patients and Controls

Twenty patients who were consecutively diagnosed with ERA [26] and had never received glucocorticoids or DMARDs (biological or nonbiological) were recruited from the hospital's Early Arthritis Clinic over a 2-year period. At the time of enrolment, all the patients had an active disease. The control group included 20 age- and sex-matched blood donors recruited during the same period. Patients and controls were excluded if they had previously diagnosed cardiovascular disease, renal disease, dyslipidemia, and/or diabetes. 

### 2.2. Treatment and Duration of Followup

Patients in the ERA group were started on conventional DMARD therapy plus glucocorticoids and followed in the Early Arthritis Clinic. Responses were evaluated after 3 months, and if there was no decrease in the 28-Joint disease activity score (DAS28), the patient was switched to biological TNF*α* inhibitor therapy. Followup ended after a total treatment period of 12 months. 

### 2.3. Assessments

A 20 dL sample of peripheral venous blood were collected at baseline from each ERA patient and control. Erythrocyte sedimentation rates were determined with standard procedures. Serum was isolated by centrifugation (3000 ×g for 10 min at room temperature), aliquoted, and stored at −80°C before analysis. 

Serum levels of ADMA, apelin and anticyclic citrullinated peptide (aCCP) antibody titers were determined with commercial human enzyme linked immunosorbent assay (ELISA) kits used according to the manufacturers' instructions (ADMA ELISA kit, Vinci Biochem—Florence, Italy; Apelin ELISA kit, Phoenix Pharmaceuticals, Inc., CA, USA; anti-CCP2 ELISA kit, Axis-Shield—Dundee, UK). Each assay was performed in duplicate, and the mean result ± SE is reported. For each ERA patient, we also recorded the number of tender/swollen joints and patient's global health assessment were recorded for the disease activity assessment performed using the DAS28 [27]. All of the above-listed assessments were repeated at the end of followup. 

All patients underwent echo-color Doppler of the carotid arteries to evaluate IMT. The carotid arteries were evaluated at baseline and followup with high-resolution B-mode ultrasonography (Esaote Biomedica, MyLab Vinco). One longitudinal image of the common carotid artery and 3 longitudinal images of the internal carotid artery were acquired. The maximal IMT of the common carotid artery and of the internal carotid artery was defined as the mean of the maximal IMT of the near and far walls on both the left and right sides. Carotid IMT was defined as a composite measure that combined the maximum common and internal carotid wall thickness of the left and right carotid arteries after standardization [28].

The study protocol was approved by the Institutional Review Board of the Policlinico Umberto I (Sapienza University of Rome), and written informed consent was obtained from all participants.

### 2.4. Statistical Analysis

Data for matched pairs were analyzed with the Wilcoxon signed-rank test. Correlations were evaluated with the Spearman rank correlation test. A probability (*P*) value of 0.05 was considered significant.

## 3. Results

### 3.1. Clinical Characteristics of ERA Patients

The clinical characteristics of the 20 ERA patients at baseline are shown in Table 1. All 20 were started on glucocorticoids and DMARDs therapy, and 14 continued these drugs for the duration of the 12-month observation period: methotrexate (MTX, 15 mg/week) alone (*n* = 7) or MTX with either hydroxychloroquine (400 mg/day) (*n* = 3) or sulfasalazine (1.5 g/day) (*n* = 1). The other 6 were treated for the first 3 months with one of the regimens listed above, but no response was observed, so they were switched to the biological TNF*α* inhibitor, adalimumab. All 20 ERA patients received glucocorticoids (mean daily dose: 7.5 mg) for the entire 12-month period.

As shown in Table 1, the 12-month follow-up visit revealed a significant decrease (versus baseline values) in the mean DAS28 (*P* = 0.0006). A reduction in RF- and/or aCCP-positivity was also observed.

### 3.2. Serum Levels of ADMA and Apelin

**Figure 1:**
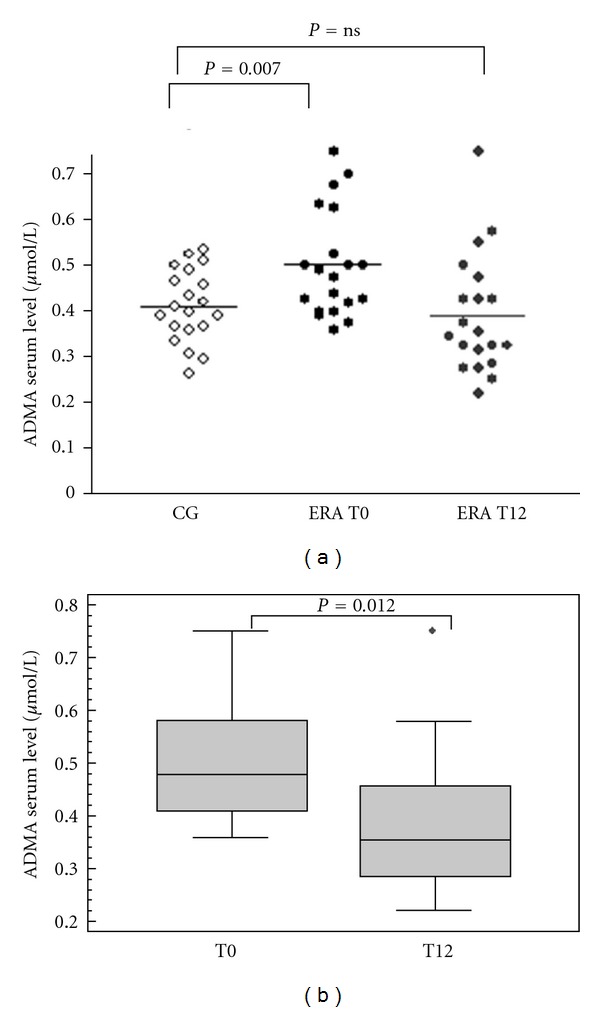
ADMA serum levels in ERA patients and controls (a) and in ERA patients before and after treatment (b). CG: control group; ERA: early rheumatoid arthritis.

**Figure 2:**
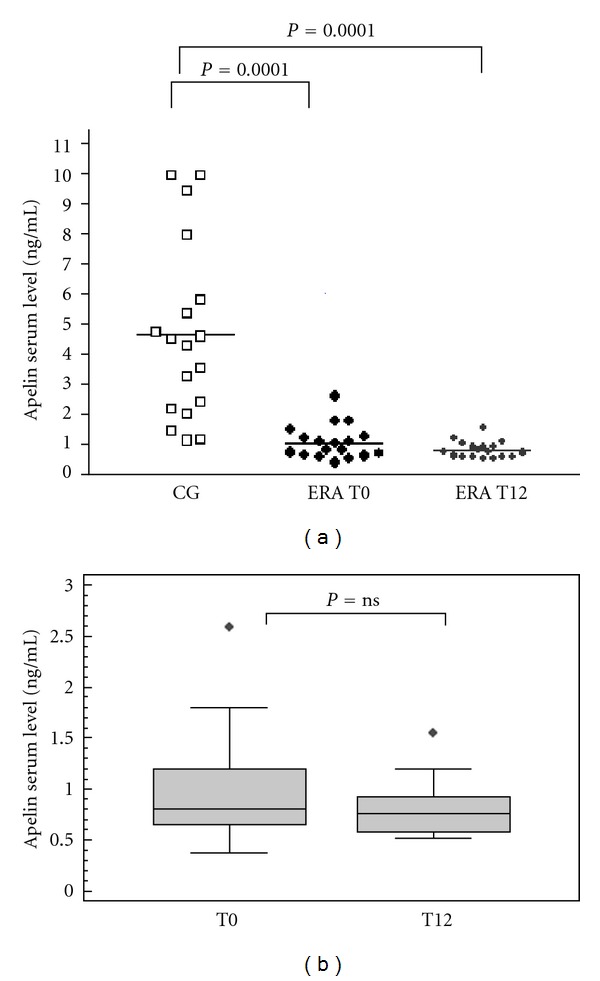
Apelin serum levels in ERA patients and controls (a) and in ERA patients before and after treatment (b).

At baseline, the mean serum ADMA level for the ERA group was significantly higher than that of controls (0.55 ± 0.03  *versus*  0.41 ± 0.02  ±  *μ*mol/L, *P* = 0.0070), but after 12 months of treatment, the mean level for these patients was significantly lower (0.38 ± 0.03 *μ*mol/L; *P* = 0.012 versus baseline) and was no longer different from the control value (*P* > 0.05) (Figure 1). As for serum apelin, the ERA group had significantly lower mean levels than controls at baseline (1.06 ± 0.56 versus 4.67 + 3.0 ng/mL, *P* = 0.0001) and at the 12-month follow-up visit (0.81 ± 0.27 versus 4.67 + 3.0 ng/ml, *P* = 0.0001). In this case, the decrease observed after 12 months of treatment was appreciable but not statistically significant.  Figure 2 shows apelin levels in RA patients and control group.

Neither of the potential markers of endothelial function displayed significant correlation with DAS28, swollen/tender joint counts, ESR, CRP, RF, or aCCP antibody titers. 

### 3.3. Echo-Color Doppler of the Carotid Arteries

Mean IMT values at baseline was 0.73 ± 0.15. After 12 months of treatment, mean IMT was 0.73 + 0.14 (Table 1). No significant difference from baseline to followup was observed.

## 4. Discussion

The increased risk of cardiovascular disease observed in RA can be attributed to accelerated, early atherosclerosis. Using Doppler ultrasound techniques, several groups have documented impaired flow-mediated dilatation (FMD) and IMT in patients with longstanding RA despite chronic DMARDs treatment [29–32]. Moreover, an association with the shared epitope has been detected, suggesting that HLA-DRB1 allele status may predict cardiovascular risk in these patients [32].

In a recent study by Södergren et al. on 79 patients with newly diagnosed RA, no signs of early atherosclerosis were detected since FMD and IMT values did not differ between ERA and control group [33]. Endothelial function in ERA patients was investigated only in other two previous, smaller studies. Bergholm et al. [15] found impaired FMD in 10 ERA patients, which improved after 6 months of therapy, and more recently, similar results were reported by Hannawi et al. in patients who had been treated for 1 year [34]. In contrast with the Swedish registry, other authors detected evidence of subclinical CVD in ERA patients who showed a higher prevalence of increased IMT of the carotid arteries and atherosclerotic plaques compared with controls [13, 14].

Doppler ultrasonography is the method most widely used to evaluate early atherosclerotic modification of arterial wall (i.e., impaired endothelial function and intima-media thickening) and it has several advantages, including noninvasiveness, widespread availability, and relatively low cost. However, while IMT expresses a morphological change of the arterial wall which increases with disease progression becoming more evident in longstanding RA, brachial FMD represents an impaired endothelial responsiveness which indicates a distinct and independent stage of atherosclerotic process. These surrogate markers of atherosclerosis seems to poorly correlate, particularly in the early stage of the disease. This statement has been confirmed in a recent study on 118 RA patients in which no correlation between FMD and carotid IMT has been detected in patient with less than 7 years of disease, but an inverse correlation become apparent in patients with longer disease duration [35]. 

One of the major limitations of ultrasonographic investigation of IMT and FMD is substantial operator-dependency. For this reason, it is important to identify other markers of endothelial function in ERA patients, which are less susceptible to this type of variability. As candidate markers, we evaluated serum levels of ADMA and apelin, which modulate NO homeostasis by inhibiting (ADMA) or activating (apelin) eNOS [24, 36]. 

Elevated serum ADMA levels have been associated with several inflammatory states and several mechanisms have been proposed to explain this link, including downregulation of dimethylarginine dimethylaminohydrolase activity as a result of oxidative stress induced by proinflammatory cytokines [37], increased expression of protein arginine type I *N*-methyltransferase, which is responsible for ADMA synthesis [38], and increased endothelial cell turnover with potential liberation of ADMA during cell catabolism [36]. In our cohort of treatment-naive ERA patients, baseline ADMA levels were significantly higher than control values, and they returned to the normal range after 12 months of conventional or biological DMARD therapy. These findings suggest that early intervention in RA might help to restore endothelial function. With respect to this, different anti-TNF agents have proved to improve endothelial function in RA patients refractory to conventional therapy [39, 40]. In their recent study of endothelial function in ERA patients, Turiel et al. [41] found that DMARD therapy had no effect on ADMA levels although it did improve 2D-echo-derived coronary flow reserve (CFR), which is indicative of at least partial restoration of vascular function. To explain the discrepancy between ADMA and CFR modification, the authors hypothesized that RA treatment could affect vascular function throughout pathways different from NO cycle. The absence of effects on ADMA levels contrasts with our findings in the ERA group, but it is also inconsistent with recent findings reported by Kuwahata et al. [42], who found that acetylcholine-induced increases in coronary blood flow are inversely correlated with ADMA levels, at least in women. The discrepancies between the results of these studies may be largely due to differences in the patient enrolment criteria, observation times, and above all the small size of the cohort studies. 

As for apelin, this recently characterized adipokine [43] has been shown to induce vasodilatation by activating eNOS [25] and its effect is reduced by eNOS inhibition [44]; apelin has been demonstrate to act as a coronary vasodilator and, when administered at systemic doses, reduces peripheral vascular resistance [44]. High serum levels would be expected to have an antiatherogenic role improving endothelium-dependent vasorelaxation; in murine models, apelin has been shown to increase vascular nitric oxide generation and reverses endothelial dysfunction [45] and to reduce macrophage infiltration into the arterial wall by direct anti-inflammatory effect within the vessel wall [46]. Low apelin levels have been detected in patients with high LDL levels [47] and those with type 2 diabetes mellitus [48], both of which are associated with an increased risk for atherosclerosis. Apelin levels have also been shown to correlate with levels of the adhesion molecules VCAM-1 and E-selectin [49]. The ERA patients we investigated presented low baseline levels of apelin along with elevated ADMA levels, which adds support to the hypothesis that endothelial dysfunction in these patients is related to altered NO homeostasis. Unlike ADMA levels, however, serum apelin levels were not significantly affected by treatment in our ERA group. This discrepancy suggests that apelin and ADMA may be independent indices of endothelial function with different degrees of sensitivity. It is important to recall that apelin is thought to exert a wide range of effects on different organs, and although its role in cardiovascular diseases is well established, its exact effect on endothelial cells has not been clearly defined. In addition, apelin is an adipokine, and its expression can be modulated by steroids. It may also be involved in immune and neurohormonal signaling [50]. It follows that apelin metabolism in RA patients is probably influenced by multiple factors.

As previously described by others [33, 41], our patients did not show any increase in carotid IMT; nevertheless, carotid IMT is a marker of structural damage of arterial wall reflecting the chronic atherosclerotic process. It is still debated if inflammation could have a rapid impact on vessel structure as assessed by IMT or the detection of earlier, preclinical and reversible atherosclerotic change such as endothelial dysfunction could be more affected by inflammatory state and its treatment in RA patients [51]. 

Finally, RA treatment reduces the inflammatory state as demonstrated by the reduction of disease activity. In our ERA patients, the DAS28 was significantly lower after 12 months of traditional and/or biological DMARD therapy, but this parameter showed no correlation with serum ADMA nor apelin levels. This correlation has also failed to emerge from previous case-control studies, probably because of the small number of patients included [22, 23, 37]. Even if in our study patients with hypercholesterolemia were excluded, a limit could be the lack of complete lipid profile at baseline and followup; however, other studies failed to demonstrate any correlation between ADMA and HDL cholesterol [23]. In this way, it would be interesting to investigate on larger groups of patients the correlation of endothelial function with inflammation-related cardiovascular markers.

This small, prospective study has been designed to assess the effect of RA treatment on two endothelial biomarkers ADMA and, for the first time apelin. The major shortcoming of our study is the size of the cohort we examined. Future attempts to address this issue should involve much larger populations for a longer followup.

In conclusion, it is reasonable to speculate that early, aggressive treatment of RA aimed at suppressing the inflammatory response and inducing disease remission might reduce the progression of endothelial damage. Serological markers and Doppler ultrasonography could both be used to assess endothelial function in patients with early-stage RA, not only for the purpose of detecting existing impairment but also for estimating the risk of cardiovascular disease.

## Figures and Tables

**Table 1:** Demographic and clinical characteristic of ERA patients.

F/M	13/7
Age, yrs	51 ± 14.2 (27–77)
Mean ± SD (range)	
Disease duration, months	7.5 ± 9.5 (1–24)
Mean ± SD (range)	
DAS28 baseline	5.0 ± 2.9 (2.39–8.58)
Mean ± SD (range)	
DAS28 followup	2.9 ± 1.5 (0.84–5.86)^ ∗^
Mean ± SD (range)	
IMT baseline, mm	0.73 ± 0.15 (0.44–0.80)
Mean ± SD (range)	
IMT followup, mm	0.73 ± 0.14 (0.45–0.90)
Mean ± SD (range)	
Corticosteroids	20/20
Methotrexate	14/20
Hydroxychloroquine	4/20
Sulfasalazine	3/20
Adalimumab	6/20
Etanercept	0/20

ERA: early rheumatoid arthritis; DAS28: disease activity score 28.

^∗^ *P  *<0.05 versus baseline value.
